# Maternal Mental Health following Ultrasonographic Detection of Fetal Structural Anomaly in the Midst of the COVID-19 Pandemic

**DOI:** 10.3390/ijerph182412900

**Published:** 2021-12-07

**Authors:** Nur Rowaidah Roslan, Mohd Fadhli Mohd Fauzi, Lim Wan Teng, Abdul Ghani Nur Azurah

**Affiliations:** 1 Department of Obstetrics and Gynaecology, Faculty of Medicine, Universiti Kebangsaan Malaysia, Jalan Yaacob Latiff, Bandar Tun Razak, Cheras, Kuala Lumpur 56000, Malaysia; nr1504@gmail.com; 2 Department of Obstetrics and Gynaecology, Hospital Tunku Azizah, Kuala Lumpur 50586, Malaysia; 3 Cheras District Health Office, Jalan Yaacob Latiff, Bandar Tun Razak, Cheras, Kuala Lumpur 56000, Malaysia; fadhli16288@yahoo.com; 4 Department of Obstetrics and Gynaecology, Hospital Tengku Ampuan Rahimah, Jalan Langat, Klang 41200, Malaysia; tengwan@yahoo.com

**Keywords:** stress, anxiety, depression, mental health, fetal structural anomaly, COVID-19, pandemic, ultrasound, Malaysia, repeated measures ANOVA

## Abstract

Prenatal ultrasonographic detection of fetal structural anomaly may adversely affect maternal mental health throughout pregnancy, particularly in the current COVID-19 pandemic. This study aims to prospectively assess maternal stress, anxiety, and depression following ultrasonographic detection of fetal structural anomaly from diagnosis until delivery during the COVID-19 pandemic. A total of 141 pregnant women at a tertiary hospital who underwent detailed scans between 16 and 24 gestational weeks were included and categorized into the study (anomaly finding, *n* = 65) and comparison (normal finding, *n* = 76) groups. Self-administered questionnaires of 10-item Perceived Stress Scale (PSS-10) and Hospital Anxiety and Depression Scale (HADS) were used to assess maternal stress, anxiety, and depression at prior detection (T_1_), two-to-four weeks post-detection (T_2_), one-to-two weeks prior to delivery (T_3_), and one-to-two weeks post-delivery (T_4_). Repeated measures of analysis of variance (ANOVA) were conducted to assess time-, between-group, and time–group interaction effect. In general, maternal stress improved, but anxiety worsened, while depression persisted, over the time from T_1_ to T_4_. The average maternal stress and anxiety levels were significantly higher among groups with fetal anomaly. The maternal stress and anxiety level were significantly affected within one-to-two weeks post-detection of fetal structural anomaly. In conclusion, maternal mental health parameters were affected differently during the COVID-19 pandemic, with higher vulnerability of stress and anxiety among pregnant women with fetal structural anomaly particularly within one-to-two weeks post-detection.

## 1. Introduction

Maternal mental health disorders are the commonest illness related to pregnancy with considerable adverse impacts against mother, fetus/children, and economy [1]. Prior to pandemic, the prevalence of antenatal and postnatal anxiety disorder has been reported at 15% to 20% and 10%, respectively [2,3], while the prevalence of depression is 7% to 25% [4,5]. Maternal mental health disorders might be under-detected and/or under-treated during pregnancy [1,6,7,8]. For instance, history taking on mental health such as anxiety about outcome of pregnancy is not usually explored during visit [9]. These under-detected and under-treated issues are critical as early psychological and psychosocial interventions have been shown to be effective and cost-effective [10,11]. Notwithstanding, pregnant women are still considered therapeutic orphans [12], and therefore they must be mainstreamed into healthcare research and practice, more so during this era of the COVID-19 pandemic [12,13].

In the midst of the COVID-19 pandemic, it is not unexpected that people are at risk of adverse mental health conditions due to an abrupt, significant changes in physical, social, and spiritual life [14]. It has been reported a pooled prevalence of 25.6% and 30.5% for depression and anxiety, respectively, during the COVID-19 pandemic [15], which are higher than prior to the pandemic [2,3,4,5]. A recent longitudinal study reported that pregnant women had a more pronounced increase in anxiety and depression during the pandemic as compared to non-pregnant women [16]. As compared to a prior pandemic, pregnant women also had higher prevalence of moderate and severe depression [16] which is also similarly reported by multiple studies during the pandemic [17,18,19,20,21]. Nonetheless, mental health is often regarded less important than physical ones during the pandemic [22]. Apart from physical health, it is in fact necessary to promote maternal mental health and the capacity on mental health care services [23], particularly in a more vulnerable group of pregnant women. However, there is currently limited study on identifying higher risk group of having mental health disorders among pregnant women to prioritize the services.

The COVID-19 pandemic significantly affects pregnancy-related healthcare. For instance, pregnant women experience difficulties accessing the antenatal services due to lockdown measures, transportation disruptions, or reluctant to come to health facilities due to fear of infection [24]. In addition, expectant mothers may have to go through a higher level of anxiety and stress as no spouse or companion are allowed during antenatal visit, or at delivery ward and delivery suite to support them [25]. Furthermore, diversion of resources and disruption of maternity services to prioritize the COVID-19 response increase risks of maternal morbidity and mortality [24]. Ilska et al. (2021) highlighted that the pandemic disrupted obstetric appointments such as the regularity of appointments, availability of medical care in obstetric-related emergent situations, and involvement in antenatal classes [26]. Likewise, similar difficulties in prenatal care happen in Malaysia [27]. It has been reported that 22.4% of pregnant women perceived that their antenatal appointments were affected, 34.7% felt anxious when their spouse was not allowed to accompany during antenatal visit, 20.7% were worried about having to give birth during the lockdown, 23.6% received unclear advice on where to deliver, and 19.8% felt worried that their spouse cannot accompany them during childbirth [27].

This current study focuses on the mental health following ultrasonographic detection of fetal structural anomaly during the COVID-19 pandemic. Fetal structural anomaly is a type of congenital anomaly which can be detected through a detailed scan prior to 24 gestational weeks among high-risk pregnant women such as advanced maternal age and family history of birth defects or genetic disorder [28]. This prenatal ultrasonographic detection may improve the chances of survival with appropriate antenatal and postnatal preparation; however, it is not without risk to maternal mental health. Being recommended for a detailed scan due to high-risk pregnancy is generally a stressful life event [29], let alone being diagnosed with a fetus with structural anomalies, which may adversely influence maternal mental health [30,31,32]. Poor mental health during pregnancy is harmful as it is associated with shorter gestation, lower birth weight, adverse neurodevelopment, and postpartum depression [29,33,34,35,36]. Multiple studies have shown that maternal mental health was affected upon prenatal detection of fetal anomaly. For instance, a prospective study in the United States found that participants with fetal anomalies that require future surgery had a greater mean state anxiety score than those without [32]. Another prospective study conducted among conveniently-sampled pregnant women who were recruited in 2006 to 2009 found that pregnant women with detected fetal structural anomaly had higher psychological distress score post-detection which gradually decreased over time [37]. Similarly, another prospective study conducted between 2008 and 2011 among pregnant women with non-lethal fetal anomalies found a general decrease in the state-anxiety score over time [38]. However, no similar study has been conducted during the COVID-19 pandemic.

It is thus plausible that this current COVID-19 pandemic may alter maternal mental health differently as compared to prior to the pandemic. However, there is still a limited number of studies that compares the risk of developing stress, depression, and anxiety between mothers bearing the fetal with and without anomalies, particularly in Malaysia during the COVID-19 pandemic. Therefore, this study was carried out to assess and compare the level of stress, anxiety, and depression during pregnancy in the midst of the COVID-19 pandemic among pregnant women following ultrasonographic detection of fetal structural anomaly and among women with normal ultrasound finding at four different time points during pregnancy: T_1_ (baseline at prior to detection of a fetal anomaly or a normal finding on ultrasound), T_2_ (two to four weeks after T_1_), T_3_ (one to two weeks prior to delivery until the time of delivery), and T_4_ (one to two weeks post-delivery). This local longitudinal data is important in confirming replicability of previous studies and planning interventional strategy in alleviating adverse mental health conditions associated with detection of fetal structural anomaly especially in the era of current pandemic situation.

## 2. Materials and Methods

### 2.1. Study Design

This was a prospective longitudinal observational study consisted of data collection at four time points: T_1_ (prior to detection of a fetal anomaly or a normal finding on ultrasound), T_2_ (two to four weeks after T_1_), T_3_ (one to two weeks prior to delivery until the time of delivery) and T_4_ (one to two weeks post-delivery).

### 2.2. Study Setting

This study was conducted from May 2020 until June 2021 among pregnant women who received or were recommended for detailed scan between 16 to 24 gestational weeks at Maternal and Fetal Medicine (MFM) Unit, Hospital Tunku Azizah, a tertiary hospital, in Kuala Lumpur. This timeframe corresponds to the usual practice of performing a detailed scan on other indicated pregnant women between 16 to 24 gestational weeks. If they failed to get certain profile, a repeat detailed scan was conducted after two weeks. The antenatal follow-up for the participants was similar with other pregnant women, that is every two to four weeks at health clinics. The follow-up under MFM clinic was done between four to six week for all participants, corresponds to usual practice. Data collection for time point T_1_ was conducted between May 2020 and September 2020 following the peak of the first wave of the COVID-19 pandemic in Malaysia.

### 2.3. Participants/Sampling

#### 2.3.1. Eligibility Criteria

Malaysian women indicated or referred for detailed scan at 16 to 24 gestational weeks with those aged above 18 years old and able to understand the Malay language were included as it is a national language in Malaysia. Women with known cases of psychiatric disorder and substance abuse were excluded.

#### 2.3.2. Sample Size

The sample size was calculated using G*Power 3.1.9.4. Based on the statistical tests of ‘ANOVA: Repeated measures, within factors’ with estimated medium effect size (partial eta square = 0.0588), α-error = 0.05 and power (1-β) = 0.80, the required total sample size is 61. Next, based on the statistical tests of ‘ANOVA: Repeated measures, within-between interaction’ with estimated medium effect size (partial eta square= 0.0588), α-error = 0.05, and power (1-β) = 0.80, the required total sample size is 176. Subsequently, we calculated using the test family of F tests and statistical tests of ‘ANOVA: Repeated measures, between factors’. By using Time 1 measurement of depression subscale in a previous study [37], we estimated standard deviation of 3.0, correlation among repeated measures of 0.5, number of groups = 2, number of measurements = 4, and calculated effect size of 0.2935. By using these parameters with α-error = 0.05 and power (1-β) = 0.80, the required total sample size is 94. With an estimated dropout of 10%, we aimed to achieve the minimum sample size of 67 participants for each group, or maximum total participants of 194.

#### 2.3.3. Sampling Technique

Participants were selected based on the appointment list name of the referred patient using systematic sampling after reviewing exclusion criteria. This technique is appropriate in view of impracticality to conduct a simple random sampling during each clinic day and uncertainty of attendance despite having an appointment list. About three patients were selected per each clinic each day from the MFM clinic using this technique. First a patient was selected from the first name on the list, then the next subject was selected in an interval of three. Eligible participants were approached in the MFM clinic. Women who consented were invited to complete the questionnaire.

### 2.4. Instruments and Data Collection

#### 2.4.1. Detailed Scan

Detailed scan with pre- and post-ultrasound counselling were performed by Fetal Medicine Specialist in the MFM clinic. Participants with fetal structural anomaly were classified into the study group while normal findings were classified into the comparison group. The study group received close maternal and fetal follow up throughout the pregnancy as per the standard of care. If needed, additional consultation by counsellor, psychiatrics, neonatology, or medical genetics case will be referred. Fetal anomalies were classified [37,39] into: (1) lethal or serious with no available treatment, with or without prognostic ambiguity (e.g., acrania, skeletal dysplasia with small thorax, holoprosencephaly), (2) serious with available treatment, with prognostic ambiguity (e.g., myelomeningocele with hydrocephalus, hypoplastic left heart syndrome), (3) mild to moderate severity with available treatment, often with good result, but with prognostic ambiguity (e.g., bilateral clubfoot or cleft lip with no other markers, condition known to be associated with syndromes not apparent prenatally), (4) mild to moderate severity with available treatment, often with good result, without prognostic ambiguity (e.g., gastroschisis, unilateral clubfoot), and (5) severity not classified; awaiting clarification.

#### 2.4.2. Clinical Record

Clinical records containing data such as parity and in-vitro fertilization (IVF) pregnancy status were extracted during T_1_ data collection.

#### 2.4.3. Self-Administered Questionnaires

Self-administered questionnaires were used to collect: (1) sociodemographic data, (2) perceived stress level using 10-item Perceived Stress Scale (PSS-10), and (3) perceived anxiety and depression level using Hospital Anxiety and Depression Scale (HADS).

The PSS-10 questionnaire is a 10-item scale that has been validated to measure the perception of stress [40]. Individual scores can range from 0 to 40 in which higher scores indicate higher perceived stress. By category, scores ranging from 0 to 13, 14 to 26, and 27 to 40 were considered low, moderate, and high perceived stress, respectively.

The HADS questionnaire is a valid and reliable 14-item scale containing two subscale measuring anxiety and depression [41,42,43,44]. For each subscale, the individual scores can range from 0 to 21 in which higher scores indicate higher anxiety and depression. By category, the total score of 0 to 7, 8 to 10, and 11 to 21 indicate low, moderate, and high levels of anxiety and depression, respectively.

### 2.5. Data Analysis

SPSS (Statistical Package for Social Science) version 25 was used for data analysis. Participants’ profiles were presented descriptively in terms of frequency and percentage, or mean and standard deviation, or median and interquartile range, depending on type and distribution of data. Bivariable analysis was conducted to compare participants’ profile between two groups using chi-square or students’ *t*-test depending on type of data. Repeated measures ANOVA was conducted for hypothesis testing in terms of time effect (T_1_–T_4_), group effect (study group vs. comparison group), and time–group interaction effect. The significance level was set at *p* < 0.05.

### 2.6. Ethical Consideration

This study is registered under the National Medical Research Register (NMRR-20-1695-55475) in Malaysia. Ethical approval has been obtained from the Medical Research and Ethics Committee (KKM/NIHSEC/P20-1709(11)). Participation is voluntary, and participants may withdraw or decline to answer any questions at any time. The ultrasound examination was performed by a Fetal Medicine Specialist in the MFM clinic to minimize the risk of misdiagnosis. Prior and after the examination, all participants were counselled by a Fetal Medicine Specialist. Both groups were given maternal and fetal follow up throughout pregnancy as per the standard of care. Referrals to the counsellor, psychiatrist, neonatologist, or medical genetics were made if necessary.

## 3. Results

### 3.1. Participants’ Profile

A total of 158 participants were initially included which were divided into the study group (*n* = 69) and the comparison group (*n* = 89). However, only 141 participants completed the whole study. Throughout the four data collection points, there were dropouts from the study group (*n* = 4) and the comparison group (*n* = 13). Reasons for dropout in both groups were withdrawn from the study (*n* = 2), did not attend any one assessment (*n* = 6), or were lost to follow up (*n* = 9). The final number of participants were 65 and 76 from the study group and the comparison group, respectively. The severity of fetal anomaly among study groups (*n* = 65) were as follows: category 1 (*n* = 15), category 2 (*n* = 1), category 3 (*n* = 1), category 4 (*n* = 6), and category 5 (*n* = 42). Table 1 showed the overall participants sociodemographic and clinical profile. The mean age of participants was 31.15 (SD = 5.506) years.

Table 2 demonstrates baseline differences according to groups. There were no significant differences between groups in terms of age, ethnicity, education level, and IVF pregnancy status.

### 3.2. Participants’ Mental Health Profile

Table 3 demonstrates the participants’ mental health profile with statistical differences between the groups at each time point. There was no significant difference in the baseline maternal stress (t = −0.074; df = 139; *p* = 0.941), anxiety (t = 0.243; df = 139; *p* = 0.808), and depression (t = −1.224; df = 139; *p* = 0.223) level between groups at T_1_. However, the level of stress and anxiety were significantly higher among the study group as compared to the comparison group at T_2_, T_3_, and T_4_. On the contrary, the level of depression was significantly higher among comparison group at T_4_ despite no significant difference between groups at T_2_ and T_3_.

The between-group effect with repeated measure ANOVA found that, at level T_2_–T_4_. the average maternal stress (mean difference = 2.027; F = 8.373; df = 1; *p* = 0.004) and anxiety (mean difference = 1.290; F = 18.151; df = 1; *p* < 0.001) were significantly higher among the study group as compared to the comparison group. However, it was found that there was no significant difference in the average maternal depression level at level T_2_–T_4_ between the two groups (F = 3.442; df = 1; *p* = 0.066).

**Figure 1:**
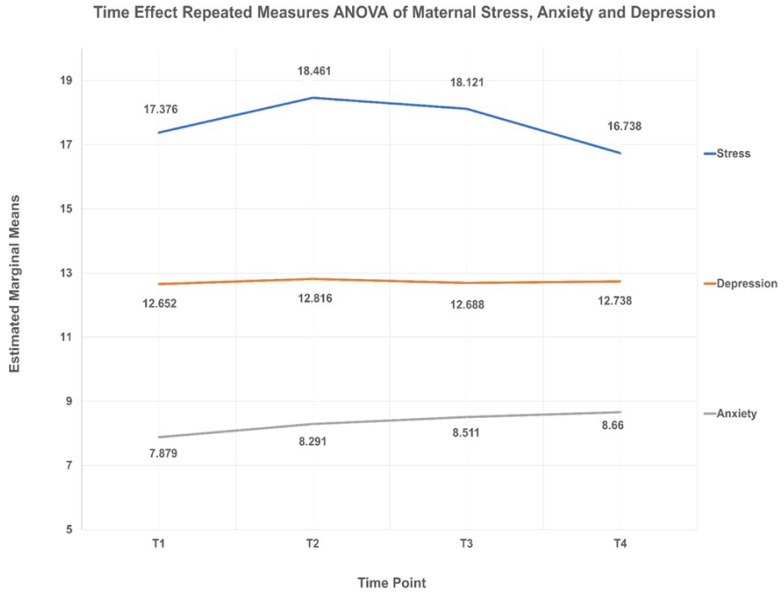
Time effect repeated measures ANOVA of maternal stress, anxiety, and depression. Note: (1) A stress score between 14 and 26 indicates moderate stress, (2) An anxiety score between 0 and 7 and between 8 and 10 indicate low and moderate levels of anxiety, respectively, (3) A depression score between 11 and 21 indicate high levels of depression.

Figure 1 illustrates the time-effect repeated measures ANOVA of maternal stress, anxiety, and depression. Despite persistent moderate level of maternal stress (score 14 to 26), there was a significant reduction of maternal stress level (F = 6.417; df = 1; *p* < 0.001) between T_2_ and T_4_ (mean difference = 1.723; 95% CI= 0.465, 2.982; *p* = 0.002) and between T_3_ and T_4_ (mean difference = 1.383; 95% CI = 0.423, 2.343; *p* = 0.001). On the contrary, there was a significant increment of maternal anxiety level (F = 7.240; df = 1; *p* < 0.001) between T_1_ and T_2_ (mean difference = −0.437; 95% CI= −0.828, −0.047; *p* = 0.019), T_1_ and T_3_ (mean difference = −0.682; 95% CI= −1.162, −0.202; *p* = 0.001), and T_1_ and T_4_ (mean difference = −0.845; 95% CI= −1.356, −0.334; *p* < 0.001) from low (T_1_) to moderate level (T_2_, T_3_, and T_4_). On the other hand, there was no significant changes in maternal depression level (F = 0.312; df = 2.698; *p* = 0.795) over time which remains at high level.

**Figure 2:**
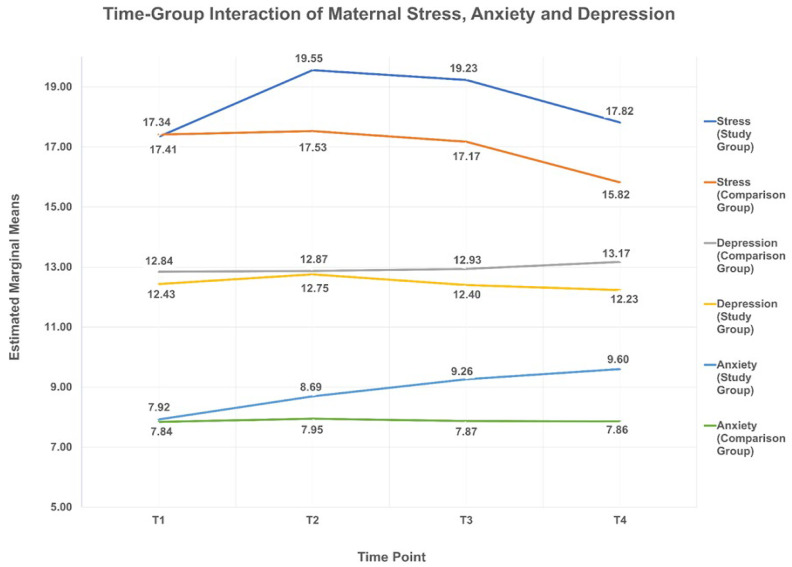
Time–group interaction of maternal stress, anxiety, and depression. Note: (1) A stress score between 14 and 26 indicates moderate stress, (2) An anxiety score between 0 and 7 and between 8 and 10 indicate low and moderate levels of anxiety, respectively, (3) A depression score between 11 and 21 indicate high levels of depression.

Figure 2 illustrates the time–group interaction effect repeated measure ANOVA. There is a significant difference in the pattern of maternal stress (F = 5.768; df = 1; *p* = 0.018) and anxiety (F = 5.177; df = 1; *p* = 0.024) level between the two groups for the T_1_–T_2_ period, in which the difference between groups at T_2_ is significantly larger than the difference between groups at T_1_. However, the same analysis found that there is no significant difference in the pattern of maternal depression level (F = 1.828; df = 2.717; *p* = 0.147) between the two groups.

A sub-analysis among participants with fetal structural anomaly divided into a group of lethal defects without treatment options while the rest were in the other group was conducted. A Students’ *t*-test analysis for each mental health parameters at each time point revealed no significant differences between group at each time point except for anxiety at T3 (prior to delivery) (mean difference = 1.917; 95% CI = 0.713, 3.121; *p* = 0.02) in which those in the lethal anomaly group recorded higher mean score of anxiety (score of 10.73) as compared to the other group (score of 8.82). Nonetheless, both scores are still within range of a moderate level of anxiety.

## 4. Discussion

We presented the first Malaysian study that assesses the level of stress, anxiety, and depression among pregnant women following ultrasonographic detection of fetal structural anomaly in the midst of the COVID-19 pandemic. Among all participants, maternal stress improved, whereas the anxiety increased over time; however, there was no change in maternal depression level over time. The maternal stress and anxiety levels were significantly higher among groups with fetal structural anomaly. The maternal stress and anxiety level were significantly affected within one-to-two weeks post-detection of fetal structural anomaly. Among participants with fetal structural anomaly, those with lethal defects recorded higher score of anxiety prior to delivery as compared to others.

Our study enhances the body of knowledge that stress, anxiety, and depression are conceptually different among each other, and can coexist during pregnancy more so during the pandemic. Our study demonstrates that overall maternal stress improved, but anxiety worsened, while depression persisted, over time from T_1_ (prior detailed scan) to T_4_ (post-delivery) among all participants. Similar findings were found by Kaasen et al. (2017) prior to the pandemic in which there was an improvement of maternal psychological distress, but persistent depression levels over time following ultrasonographic detection of a fetal anomaly [37]. However, two prospective studies among pregnant women recruited around 2006 to 2011 reported opposite findings on the trend of maternal anxiety as compared to our study [37,38] which could probably be due to differential methods such as instruments and recruitment time. In addition, we postulated that the worsening anxiety and persistently high level of depression could also be due to the challenges faced by pregnant women in the midst of the COVID-19 pandemic [45,46,47,48] whereby there was difficulty in getting clinic appointments and the restriction of not being accompanied by their spouse during antenatal visit [49].

Our study confirmed that being diagnosed with fetal structural anomaly puts the mother at higher risk of stress and anxiety despite a similar baseline level. This finding is in line with other studies prior to the COVID-19 pandemic [39,50]. It is plausible that being told that current pregnancy is indicated for detailed scan is a stressful life event, and the communication of diagnosis of a fetal anomaly can be a psychological trauma to the mothers [51]. Poor mental health conditions may subsequently be harmful for the health of the mother and fetus [29]. Hence, it is important that pregnant women with ultrasonographic detection of fetal structural anomaly be given priority for psychological intervention throughout pregnancy [52] including during the current pandemic.

Our study also highlights that the prenatal ultrasonographic detection of fetal structural anomaly largely affects maternal stress and anxiety level as early as one-to-two weeks post-detection. Similar findings were found by Wilpers et al. (2017) in which maternal anxiety state level was higher among mothers with fetal anomaly [32]. Similarly, Kaasen et al. (2017) reported an initial high level of distress post-detection of fetal anomaly but did not evaluate the baseline level [37]. This is in line with the general psychological response following traumatic events [51]. This finding reflects that the intervention following fetal anomaly detection may be offered as early as possible from prior detection to two weeks post-detection as this is the significant time period of surge in maternal stress and anxiety.

Among participants with fetal structural anomaly, those with lethal defects recorded a higher score of anxiety prior to delivery as compared to others. Previous study demonstrated that those pregnant women with lethal fetal anomaly had higher distress and depression as compared to others [39]. However, no study related to anxiety was found. We postulated that this could be contributed by the worrying feeling of going to deliver a lethal fetus as compared to a lethal one, and other outcomes related to grief of spouse or acceptance of family members with the upcoming issues post-delivery.

It is unarguable that mental health disorders among pregnant women particularly those with a diagnoses of fetal structural anomaly should be given medical attention. First, pregnant women with fetal structural anomaly have higher priority for intervention as compared to normal pregnancy. Second, the intervention should be offered as early as possible given that the worsening of mental health parameters occurred as early as one-to-two weeks post-detection. Third, multidisciplinary involvement should be practiced to intervene this issue in which both obstetrician and psychiatrist should work together to prevent, diagnose, treat, and keep safe pregnant women with mental disorders [53]. Fourth, resilience of antenatal and postnatal care must be enhanced, and pandemic should not be an excuse to provide sub-standard care [54]. For instance, an investment into telemedicine or virtual mental health consultation must be considered to tackle this issue without compromising effort to break the chain of COVID-19 infection [54].

There are several limitations of the study. First, this study was conducted among pregnant women indicated for detailed scans at 16 to 24 weeks, and therefore cannot be generalized to all pregnancies. Second, this study was conducted at a tertiary hospital setting with adequate scope of service such as psychiatric support service and standard ultrasound device for detailed scan; thus, the result cannot be generalized to other health settings. Third, this study employed a systematic sampling that reduces the potential bias in the information and reduces the risk of favoritism; however, it is not a random sample of the total population. Fourth, sample size was considerably small as the study was conducted during the early emergence of the COVID-19 pandemic and consequently substantial decrease in appointments with a majority of the patients rescheduling and some patients missing their appointments. Fifth, this study did not control the confounding variable that may influence both groups such as family support system, home environment, childhood diversity, and coping mechanism. This study also did not consider obstetric anamneses, specific difficulties in accessing prenatal care, COVID-19’s impact on obstetric care, history of COVID-19 disease and its complications, and other COVID-19 related confounders, and hence the mental health parameters are not controlled. Nonetheless, this study represents the real-life situation faced by pregnant mothers indicated for detailed scan in our study setting during the COVID-19 pandemic.

## 5. Conclusions

This study concludes that maternal mental health parameters were affected differently during the COVID-19 pandemic, with higher vulnerability of stress and anxiety among pregnant women with fetal structural anomaly particularly within one-to-two weeks post-detection. It is thus important to consider providing adequate psychological support for pregnant women within two weeks post-detection of fetal anomaly, and they should be put at top priority. However, further study is needed to determine whether findings are similar if conducted at other settings and to determine the effective and efficient methods for psychological intervention among them.

## Figures and Tables

**Table 1:** Participants’ profile (*n* = 141).

Variables	Sub Variables	Frequency (*n*)	Percentage (%)
Ethnicity	Malay	118	83.70
	Non-Malay (Chinese/Indian/Others)	23	16.31
Level of Education	No formal education	6	4.30
	School/Pre-University	40	28.37
	University (Diploma/Degree/Master)	95	67.38
Marital Status	Married	138	97.90
	Separated/Divorced	3	2.10
Household Income	B_40_ (less than RM 4360.00)	93	66.00
	M_40_ (RM 4360.00 to RM 9619.00)	40	28.40
	T_20_ (more than RM 9619.00)	8	5.70
Parity	Nulliparous	40	28.40
	Multiparous	101	71.60
IVF Pregnancy Status	Yes	4	2.80
	No	137	97.20

**Table 2:** Participants’ baseline differences (*n* = 141).

Variables	Sub Variables	*n* (%) ^1^		χ^2^	df	*p*-Value
		Study Group (*n* = 65)	Comparison Group (*n* = 76)			
Age, in years	18 to 35	56 (47.5)	62 (52.5)	0.537	1	0.464
	More than 35	9 (39.1)	14 (60.9)			
Ethnics	Malay	52 (44.1)	66 (55.9)	1.201	1	0.273
	Non-Malay	13 (56.5)	10 (43.5)			
Level of Education	Non-University	24 (52.2)	22 (47.8)	1.014	1	0.314
	University	41 (43.2)	54 (56.8)			
IVF Pregnancy Status	No	64 (46.7)	73 (53.3)	0.738	1	0.390
	Yes	1 (25.0)	3 (75.0)			

^1^ Row percent.

**Table 3:** Participants’ mental health profile (*n* = 141).

Variables	Sub Variables	*n* (%)			Mean Difference	t (df)	95% CI	*p*-Value
		All (*n* = 141)	Study Group (*n* = 65)	Comparison Group (*n* = 76)				
**Stress ^1^**	**T_1_**	17.38 (5.530)	17.34 (4.960)	17.41 (6.007)	−0.069	−0.074 (139)	−1.923, 1.784	0.941
	**T_2_**	18.46 (5.944)	19.55 (4.899)	17.53 (6.600)	2.028	2.042 (139)	0.064, 3.991	0.043 ^4^
	**T_3_**	18.12 (4.554)	19.23 (4.130)	17.17 (4.709)	2.060	2.739 (139)	0.573, 3.547	0.007 ^4^
	**T_4_**	16.74 (4.788)	17.82 (4.673)	15.82 (4.721)	2.000	2.519 (139)	0.430, 3.569	0.013 ^4^
**Anxiety ^2^**	**T_1_**	7.88 (1.962)	7.92 (1.753)	7.84 (2.136)	0.081	0.243 (139)	−0.577, 0.739	0.808
	**T_2_**	8.29 (2.113)	8.69 (1.968)	7.95 (2.184)	0.745	2.112 (139)	0.048, 1.442	0.036 ^4^
	**T_3_**	8.51 (2.260)	9.26 (2.167)	7.87 (2.150)	1.393	3.821 (139)	0.672, 2.114	<0.001 ^4^
	**T_4_**	8.66 (2.390)	9.60 (2.397)	7.86 (2.083)	1.745	4.625 (139)	0.999, 2.491	<0.001 ^4^
**Depression ^3^**	**T_1_**	12.65 (1.993)	12.43 (1.936)	12.84 (2.033)	−0.411	−1.224 (139)	−1.076, 0.253	0.223
	**T_2_**	12.82 (2.212)	12.75 (2.312)	12.87 (2.138)	−0.115	−0.306 (139)	−0.856, 0.627	0.760
	**T_3_**	12.69 (1.979)	12.40 (2.022)	12.93 (1.921)	−0.534	−1.607 (139)	−1.192, 0.123	0.110
	**T_4_**	12.74 (2.086)	12.23 (2.037)	13.17 (2.042)	−0.940	−2.729 (139)	−1.622, −0.259	0.007 ^4^

^1^ Stress score between 14 and 26 indicates moderate stress; ^2^ Anxiety score between 0 and 7 and between 8 and 10 indicate low and moderate level of anxiety, respectively; ^3^ Depression score between 11 and 21 indicates high levels of depression; ^4^ Significant at *p* < 0.05.

## Data Availability

The data presented in this study are available on request from the corresponding author. The data are not publicly available due to ownership belongs to the institution where the study was conducted.
